# Effect of a Nutrition Support Formula in Adults With Inflammatory Bowel Disease: A Pilot Study

**DOI:** 10.1177/2164956119867251

**Published:** 2019-07-29

**Authors:** Jennifer J Ryan, Douglas A Hanes, Ryan D Bradley, Nikhat Contractor

**Affiliations:** 1 Helfgott Research Institute, National University of Natural Medicine, Portland, Oregon; 2 Family Medicine and Public Health, University of California, San Diego, La Jolla, California; 3 Metagenics, Inc., Aliso Viejo, California

**Keywords:** Crohn’s disease, folate, inflammatory bowel disease, neutrophils, red cell distribution width, ulcerative colitis

## Abstract

**Background:**

Due to the high prevalence of nutrient deficiencies in patients with inflammatory bowel disease (IBD), routine monitoring of nutrient status and supplementation are recommended.

**Objective:**

This preliminary study was implemented to prospectively identify potential effects of a nutrition support formula on blood nutrient parameters in adults with IBD.

**Methods:**

Ten adults with Crohn’s disease or ulcerative colitis were recruited from the Portland, Oregon, metropolitan area into a single-arm, open-label pilot study. Participants consumed a nutrition support beverage twice daily for 12 weeks. The formula contained a mixture of micronutrients (including methylated forms of folate and vitamin B12), macronutrients, and phytonutrients (including curcumin, xanthohumol, ginger compounds, and quercetin). Primary measures were the following parameters: folate, vitamin B12, red blood cell (RBC) count, hemoglobin, hematocrit, electrolytes, and albumin. Exploratory measures included a food frequency questionnaire, circulating blood cell counts, and inflammatory markers.

**Results:**

Nine participants completed the study and one withdrew. Adherence was 98%. Serum folate increased 48.7% (*P* = .029), serum vitamin B12 increased 17.4% but did not reach statistical significance (*P* = .053), and red cell distribution width (RDW) decreased 9.2% (*P* = .012) over the 12-week study period. There were minimal shifts in total white blood cell (WBC) counts (−1.0%, *P* = .845), but percent neutrophils decreased 10.4% (*P* = .042) and absolute lymphocyte count increased 18.6% (*P* = .048). RBC count, hemoglobin, hematocrit, electrolytes, albumin, and inflammatory markers did not change significantly. Post hoc analysis demonstrated that neutrophil–lymphocyte ratio (NLR) decreased 18.4% (not significant, *P* = .061).

**Conclusion:**

Serum folate and RDW improved in adults with IBD after 12 weeks. Modulation of leukocyte subtypes was also observed, including a decrease in neutrophils and an increase in lymphocytes, with no change in total WBC count. A randomized, controlled study to further examine effects of the nutrition support formula will be initiated to follow up on this promising, but preliminary investigation.

## Introduction

The inflammatory bowel diseases (IBD) ulcerative colitis and Crohn’s disease are chronic disorders characterized by inflammation of the gastrointestinal tract.^1^ Malnutrition is highly prevalent in patients with IBD and can be caused by malabsorption, inadequate dietary intake, increased rate of protein turnover, intestinal inflammation, and losses related to medications.^2–5^

Anti-inflammatory and immunosuppressive agents prescribed to treat IBD have many targets including cyclooxygenase (COX), lipoxygenase (LOX), tumor necrosis factor alpha (TNFα), nuclear factor-kappa B (NF-κB), and dihydrofolate reductase (DHFR).^6,7^ However, these medications do not address malnutrition and several may contribute to nutrient deficiencies and anemia. For example, use of the DHFR inhibitor methotrexate in patients with IBD can deplete folate and cause hyperhomocysteinemia.^8,9^ Sulfasalazine impairs folate absorption and can cause hemolytic anemia.^4,10,11^ Corticosteroids increase net protein loss.^3^ Due to the high prevalence of clinical and subclinical nutrient deficiencies in this population, routine monitoring of nutrient status, mineral and multivitamin supplementation, and increased protein intake are recommended.^2,3^

Phytonutrients are increasingly used by patients with IBD as adjuvants to pharmaceutical interventions.^12^ For example, flavonoids are among some of the most promising phytochemicals for regulating inflammation in patients with IBD and many have similar mechanisms of action as medications used to treat IBD. Curcumin, a polyphenol from turmeric root (*Curcuma longa*), has been shown to inhibit COX, LOX, TNFα, and NF-κB.^13,14^ In preclinical studies, curcumin has also been shown to inhibit neutrophil motility and to induce neutrophil apoptosis.^14,15^ Targeting neutrophils represents a novel therapeutic strategy for IBD management, given that neutrophils play a key role in intestinal mucosal injury and neutrophil apoptosis is delayed in patients with IBD.^14,16–18^ A recent systematic review concluded that curcumin has demonstrated efficacy in symptom reduction and improvement of inflammatory indices in patients with IBD, justifying the need for further studies.^13^

Another phytochemical, xanthohumol, a prenylated chalcone from hops (*Humulus lupulus*), has been shown to inhibit COX and LOX, suppress TNFα pathways, and downregulate NF-κB activation.^19–22^ In a mouse model, xanthohumol was shown to decrease dextran sulfate sodium (DSS)-induced colitis by inhibiting NF-κB signaling.^23^ However, human-subject studies examining the effect of xanthohumol on IBD are lacking and represent a novel area of research.

This preliminary study was designed to prospectively identify potential effects of a nutrition support formula on nutrient parameters in adults with ulcerative colitis or Crohn’s disease.

## Materials and Methods

### Study Design

A single-arm, open-label pilot study to assess changes in blood nutrient levels in adults with ulcerative colitis or Crohn’s disease following 12 weeks of consuming a nutrition support formula was implemented. The study also explored changes in dietary intake, inflammatory markers, blood counts, a metabolic panel, quality of life, fecal short-chain fatty acids, and intestinal commensal bacteria. Adherence and safety were monitored throughout the study. Participants were screened for eligibility by phone and then participated in a clinical screening visit to confirm eligibility. Qualifying participants returned for a baseline visit, a 6-week mid-point visit, and a 12-week study completion visit. All study-related operations were conducted at the Helfgott Research Institute at National University of Natural Medicine (NUNM). This study was approved by the Institutional Review Board at NUNM (IRB #031516) and registered at ClincalTrials.gov (NCT02801240). Participants provided written informed consent.

### Intervention

The studied nutrition support formula was manufactured and supplied by Metagenics, Inc. (Aliso Viejo, CA, USA) in 14-serving containers. The formula contains a mixture of micronutrients (including methylated forms of folate and vitamin B12), macronutrients (protein, carbohydrates, fat, and fiber), essential amino acids, and several phytonutrients and botanical extracts including curcumin as curcumagalactomannoside (mixture of curcuminoids from turmeric root/*Curcuma longa* and galactomannans from fenugreek/*Trigonella foenum-graecum* seed fiber, standardized to 40% curcuminoids), hops/*Humulus lupulus* extract (standardized to 2.5% xanthohumol coupled to a rice protein matrix), ginger root/*Zingiber officinale* extract, rosemary leaf/*Rosmarinus officinalis* extract, and quercetin (Table S1). Participants were asked to add 2 scoops of the study formula (47 g) to water or juice (8–10 ounces) and consume it twice per day as a reconstituted beverage.

### Participants and Recruitment

Adults aged 18 to 70 years with ulcerative colitis or Crohn’s disease were recruited from the Portland, Oregon, metropolitan area. Target enrollment was 10 individuals. Recruitment approaches included online advertisements and flyers. In addition, electronic health records of consenting patients of the NUNM Health Center were queried. Potentially eligible individuals were mailed an invitation letter and recruitment flyer on a rolling basis. Exclusion criteria were as follows: currently taking the study formula or a similar product (macronutrient and micronutrient support consumed as a beverage); currently taking turmeric, curcumin, fenugreek, hops, xanthohumol, ginger, rosemary or quercetin supplements; currently receiving intravenous nutrient support; currently taking anticoagulant or antiplatelet medications; currently taking oral or intravenous antibiotic, antiparasitic, or antifungal medications; initiation of or changes to medications, supplements, an exercise regime, or a nutrition plan within 28 days prior to screening; currently participating in a weight loss program; gastrointestinal surgery within 3 months prior to screening; currently have a colostomy or ileostomy bag in place; malignancy within the last 5 years; women who were lactating, pregnant or planning pregnancy during the study period; known intolerance or allergy to ingredients in the study formula; or participating in another interventional research study within 28 days prior to screening.

### Data Collection

Fasting blood samples were obtained by venipuncture at the baseline and 12-week study completion visits. The Block Brief 2000 3-month food frequency questionnaire (FFQ), a modified version of the Block 1998 FFQ, was administered at the baseline and study completion visits.^24,25^ The Gastrointestinal Quality of Life Index (GIQLI) and the Inflammatory Bowel Disease Questionnaire (IBDQ) were administered at the baseline, mid-point, and study completion visits.^26,27^ Higher GIQLI and IBDQ scores are consistent with better quality of life.^26,27^ Participants were instructed to collect a fecal sample at home within 3 days prior to the baseline and study completion visits. Participants were interviewed for adverse events at the mid-point and study completion visits, as well as by phone between study visits. To assess adherence, participants were given paper logs to track intake of the study formula. Logs and unused study materials were returned at the mid-point and study completion visits.

### Blood and Stool Sample Analysis

Fasting blood samples were analyzed for nutrient parameters that included folate, vitamin B12, red blood cell (RBC) count, hemoglobin, hematocrit, sodium, potassium, calcium, and albumin. Inflammatory markers included high-sensitivity C-reactive protein (hs-CRP) and erythrocyte sedimentation rate (ESR). Cytokines were assayed, including TNFα, interleukin-6 (IL-6), interleukin-10 (IL-10), and interleukin-17A (IL-17A). Serum and whole blood were sent to Quest Diagnostics (Seattle, WA, USA) by courier the day of collection for folate and vitamin B12 immunoassays, a complete blood count (including RBC count, hemoglobin, and hematocrit), a metabolic panel (including sodium, potassium, calcium, and albumin), hs-CRP, and ESR. Plasma was frozen at −20°, then shipped overnight on dry ice in a single batch for cytokine analysis after the study was completed; analysis was performed by Veridia Diagnostics (Round Rock, TX, USA) using a high-sensitivity immunoassay that employs single-molecule counting technology.^28,29^ Fecal samples were shipped to Genova Diagnostics (Asheville, NC, USA) within 24 hours of collection; concentrations of short-chain fatty acids were measured using gas chromatography–mass spectrometry, and commensal bacteria were identified using semiquantitative polymerase chain reaction (PCR). All laboratories were Clinical Laboratory Improvement Amendments (CLIA)-certified.

### Questionnaire Analysis

The Block Brief 2000 3-month FFQs were analyzed by NutritionQuest (Berkeley, CA, USA). Although this FFQ can be used to assess many parameters, data analysis was limited to assessing for changes in intake of nutrients most pertinent to the nutrient parameters monitored in the study, including dietary intake of folate, vitamin B12, iron, sodium, potassium, calcium, and protein. The quality of life questionnaires, the Gastrointestinal Quality of Life Questionnaire (GIQLI), and the IBDQ were scored as previously described.^26,27^

### Data Analysis

Statistical analyses were limited to data from participants who completed the study. Continuous measures are presented as mean and standard deviation at each time point. Changes from baseline to study completion were analyzed using paired *t* tests to identify significant differences, with the exception of the quality of life measures (GIQLI and IBDQ scores), which were analyzed using a random intercept model with visit (baseline, 6 weeks, and 12 weeks) as a repeated factor. All continuous variables were investigated for assumptions of normality, prior to analysis. When outcomes showed substantial skew or outliers, sensitivity analyses were conducted using log transformations of the data, or omitting outliers, as appropriate. Some PCR data had distributions that could not be corrected even by transformations, and in this case, we confirmed *t* test results with a Wilcoxon signed rank analysis. In the case of PCR data that were outside of laboratory detection limits, the extreme detectable value was imputed for analysis. Statistical analyses were performed using SPSS v.20 software (IBM Corp., Armonk, NY).

For primary blood marker outcomes, we also recorded proportions of participants who were outside of laboratory reference ranges at either time point and planned to test significance of such changes with a McNemar’s test; however, this exercise was omitted due to few values being out of range.

## Results

### Participant Characteristics

**Figure 1.**
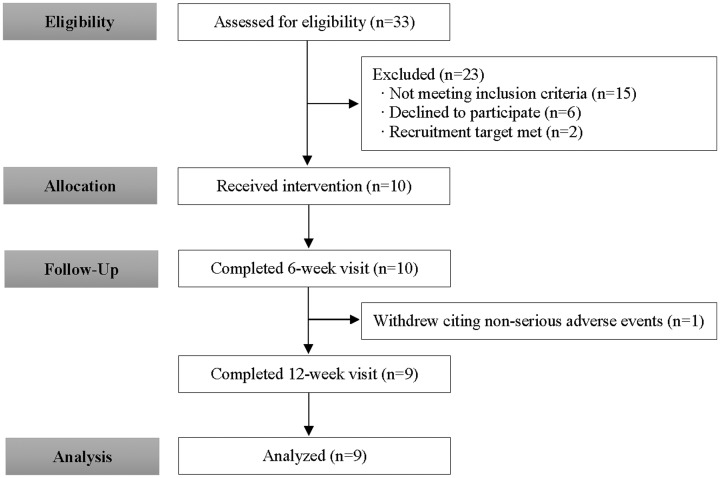
Study Flow Diagram.

Demographic parameters are described in Table 1. Flow of the study is described in Figure 1. Nine participants completed the study and 1 elected to withdraw 3 weeks before completing the study, citing nonserious adverse events (worsening of preexisting gastrointestinal symptoms). Data from the 9 participants who completed the study were analyzed.

**Table 1.** Participant Demographics at Baseline (n = 10).

	Mean ± SD or n (%)
Age (years)	39.3 ± 13.5
Body mass index (kg/m^2^)	28.3 ± 3.4
Gender	
Females	8 (80%)
Males	2 (20%)
Condition	
Ulcerative colitis	5 (50%)
Crohn’s disease	5 (50%)
IBD duration (years)	11.2 ± 6.3
Race, ethnicity	
White, not Hispanic or Latino	10 (100%)
Tobacco	
Nonuser	8 (80%)
User	2 (20%)
Alcohol use (number of beverages/week)	2.6 **±** 4.4
IBD prescription medications	
Salicylates (oral mesalamine)	4 (40%)
Purine antagonists (azathioprine, mercaptopurine)	2 (20%)
TNF blockers (adalimumab)	1 (10%)
Glucocorticoids (budesonide)	1 (10%)

Abbreviations: IBD, inflammatory bowel disease; SD, standard deviation; TNF, tumor necrosis factor.

### Adherence

Participant adherence to the study formula was quantified using 2 methods; findings were consistent between the 2 approaches and indicated that adherence was high. The weight of unused study material returned by the participants was indicative of participants consuming a mean of 98.4% of dosages (range, 85.7%–100.0%). Logs completed by the participants to track their usage of the study formula were consistent with participants consuming a mean of 97.7% of dosages (range, 90.1%–100.0%).

### Nutrient Parameters

Folate increased 48.7% (*P* = .029) over the course of the study, from 11.7 to 17.4 ng/mL (Table 2). An increase in vitamin B12 by 17.4% was not statistically significant (*P* = .053). Other primary markers showed very little change.

**Table 2.** Nutrient Parameters.

	Baseline		12 Weeks		%Δ of the Mean	*P* ^a^
	Mean	SD	Mean	SD		
Folate (ng/mL)	11.7	3.8	17.4	7.9	48.7	.029
Vitamin B12 (pg/mL)	476.7	370.8	559.6	397.8	17.4	.053
RBC count (million/µL)	4.7	0.3	4.7	0.3	0.3	.888
Hemoglobin (g/dL)	13.8	0.9	13.8	0.8	0.0	1.000
Hematocrit (%)	42.0	2.7	41.8	2.3	−0.5	.809
Sodium (mmol/L)	139.1	2.2	137.8	2.2	−1.0	.119
Potassium (mmol/L)	4.2	0.3	4.3	0.2	1.6	.455
Calcium (mg/dL)	9.4	0.3	9.5	0.3	1.1	.392
Albumin (g/dL)	4.4	0.4	4.3	0.3	−1.5	.242

Abbreviations: Δ, change; dL, deciliter; g, gram; L, liter; mg, milligrams; mL, milliliter; mmol, millimoles; ng, nanogram; pg, picograms; RBC, red blood cell; SD, standard deviation; µL, microliter.

^a^*P* values calculated using paired *t* tests.

### Dietary Intake

The current recommended dietary allowance for folate is 400 mcg dietary folate equivalents (DFE) for nonpregnant, nonlactating adults.^30^ The FFQ revealed that through diet alone, mean average daily DFE were less than the recommended 400 mcg at both time points (Table S2). The FFQ also indicated that mean daily intake of naturally occurring folate in food increased by 58.0 mcg (38.1%, *P* = .033) over the course of the study. Shifts in dietary intake of vitamin B12, iron, sodium, potassium, calcium, and protein were not significant.

### Inflammatory Markers

There were no significant changes in cytokines, hs-CRP, or ESR (Table S3). However, 1 participant who had inadvertently discontinued mesalamine (800 mg per day) approximately 1 week before the study completion visit had outlying inflammatory marker data. When their data were omitted from inflammatory marker analysis, distributions of these variables were no longer notably skewed and TNFα concentration decreased 24.5% (*P* = .016).

### Blood Counts and Metabolic Panel

On the complete blood count panel, red cell distribution width (RDW) decreased 9.2% (*P* = .012), percent neutrophils decreased 10.4% (*P* = .042), and absolute lymphocyte count increased 18.6% (*P* = .048) (Table S4). After observing the shifts in neutrophils and lymphocytes, neutrophil-lymphocyte ratio (NLR) was calculated post hoc by dividing absolute neutrophil count by absolute lymphocyte count. Mean NLR decreased 18.4% (not significant, *P* = .061, from 2.61 at baseline to 2.13 after the intervention). There were no significant changes on the comprehensive metabolic panel (Table S5).

### Quality of Life Measures and Stool Analysis

Most GIQLI and IBDQ total and subdomain scores increased from baseline to study end, but improvements were not statistically significant (Table 3). However, increases in total GIQLI score and the GIQLI Social Function and Emotional Function domain scores were more promising (*P* < .1) than shifts in IBDQ scores, which were minimal (*P* ≥ .5). Stool analysis results were unremarkable (Table S6).

**Table 3.** Quality of Life Questionnaires.

	Score Range	Baseline		6 Weeks		12 Weeks		Baseline to 12 Weeks	
		Mean	SE	Mean	SE	Mean	SE	%Δ of the Mean	*P* ^a^
GIQLI—Total	0–144	96.8	4.9	95.8	3.1	104.7	4.4	8.2	.078
GIQLI—Gastrointestinal Symptoms	0–76	54.8	2.5	53.1	2.3	58.3	2.4	6.4	.140
GIQLI—Physical Function	0–28	14.8	1.8	14.1	1.7	15.7	1.2	6.1	.491
GIQLI—Social Function	0–16	11.0	1.0	11.3	0.9	12.8	0.7	16.4	.059
GIQLI—Emotional Function	0–20	13.4	0.9	14.2	0.6	14.8	0.6	10.4	.095
GIQLI—Subjective Treatment Assessment	0–4	2.8	0.3	3.0	0.2	3.1	0.3	10.7	.245
IBDQ—Total	32–224	167.9	7.1	165.6	5.1	171.3	7.0	2.0	.661
IBDQ—Bowel Symptoms	10–70	52.1	2.7	50.1	2.0	54.6	3.0	4.8	.492
IBDQ—Systemic Systems	5–35	21.4	1.4	20.6	1.4	21.8	1.5	1.9	.812
IBDQ—Social Function	5–35	30.2	1.8	29.8	1.6	30.9	1.4	2.3	.705
IBDQ—Emotion Health	12–84	64.1	3.1	65.1	2.2	64.1	2.5	0.0	1.000

Abbreviations: Δ, change; GIGLI, Gastrointestinal Quality of Life Questionnaire; IBDQ, Inflammatory Bowel Disease Questionnaire; SE, standard error.

^a^*P* values calculated using mixed model analysis, random intercept model.

### Adverse Events

Upon query during study visits and by phone between visits, a total of 30 adverse events were documented; none were serious. Three adverse events were acute and self-limited; they included 1 upper respiratory tract infection, 1 instance of flu-like illness, and 1 instance of food-borne illness. All remaining adverse events were related to preexisting symptoms and two-thirds were gastrointestinal. The complete blood count and comprehensive metabolic panel indicated no adverse changes; mean values were within normal laboratory reference ranges at baseline and study end.

## Discussion

This study was implemented to prospectively identify potential effects of a nutrition support formula that contains micronutrients and macronutrients as well as phytonutrients that have previously been shown to affect biochemical pathways related to immune responses.^13–15,19–23^ After a 12-week course of the nutritional formula in adults with IBD, serum folate concentration increased. The formula contained 400 mcg folate per day as 5-methyltetrahydrofolate (5-MTHF). As 5-MTHF has been described as a preferred form of folate for transport into tissues (compared to synthetic folic acid) and because 5-MTHF has been shown clinically to be superior to folic acid for increasing plasma folate, this finding was not unexpected.^31,32^ However, addressing inadequate folate intake is meaningful because folate deficiency contributes to anemia, one of the most common complications and causes for hospitalization in patients with IBD.^33^ Colorectal cancer and thromboembolism are also serious sequelae of folate deficiency in patients with IBD.^5,34,35^ Therefore, improving serum folate levels is relevant and important in this clinical population.

The FFQ indicated that the study participants consumed insufficient amounts of folate through diet alone. Inadequate dietary intake is one of several factors that contribute to malnutrition in patients with IBD.^2^ The FFQ also showed that participants had consumed an average of 58.0 additional micrograms of naturally occurring folate daily over the course of the study, compared to baseline. Considering that the study formula provided an amount of folate that was 6.9 times higher than the shift in dietary intake (400 mcg vs 58.0 mcg), the improvement in serum folate is likely most attributable to the 5-MTHF in the nutritional formula.

In addition, this study demonstrated a reduction in RDW. An indicator of RBC volume heterogeneity (anisocytosis), RDW is *inversely* related to levels of folate, vitamin B12, and iron.^36^ Interestingly, several studies recently summarized by Goyal et al. indicate that RDW may have utility as a prognostic indicator of disease severity in patients with ulcerative colitis and Crohn’s disease as well as other GI disorders.^37^ Although RDW cutoffs have been proposed for distinguishing active disease from inactive disease, additional research is necessary to verify the potential of RDW as a marker of IBD severity.^38^

Additional hematologic measures yielded noteworthy findings. The decrease in neutrophils and increase in lymphocytes, along with minimal change in total leukocyte count, suggest a modulatory effect on leukocyte subtype. These findings may be related to the highly bioavailable curcumin in the formula.^39,40^ Neutrophil apoptosis has been demonstrated to be delayed in patients with IBD and curcumin has exhibited the ability to induce neutrophil apoptosis both *in vitro* and *in vivo*.^15,18^ Churchill et al. reported that curcumin increased small intestinal mucosal CD4+ T lymphocytes and B lymphocytes in mice that were treated for nearly 11 weeks.^41^ It was recently reported that curcumin increased the proportion of colonic mucosal CD4+ Foxp3+ regulatory T lymphocytes in a murine model of experimental colitis.^42^ Therefore, biologic mechanisms for the modulation of leukocyte subtypes are potentially related to the actions of curcumin. Follow-up investigations should examine this observation further to determine if specific lymphocyte subtypes are modulated by the nutritional formula or its ingredients, with curcumin being a prime target for initial experimentation.

This study also revealed an unanticipated post hoc finding related to the decrease in neutrophils and increase in lymphocytes. Mean neutrophil-lymphocyte ratio (NLR) decreased, yet did not reach statistical significance (*P* = .061). NLR has recently been described as a novel and noninvasive marker of IBD activity and severity in patients with ulcerative colitis and Crohn’s disease.^43,44^ However, additional research is necessary to confirm the utility of NLR as a marker of IBD activity.

This study has both strengths and limitations. Strengths include successful implementation of a pilot experimental design, devised to collect comprehensive data on a nutrition support formula not previously evaluated in a prospective clinical study. An additional strength was excellent adherence to the intervention by the participants. Perceived limitations may include the small sample size and not including a control group. However, according to Aickin, early-phase studies should use small sample sizes and do not always require an untreated group, since the true purpose of early-phase research is to guide future research effort.^45^

To follow up on the findings of this pilot study, a randomized, controlled study is being devised. Impact of the nutritional formula on nutrient parameters, quality of life, RDW, leukocyte subtypes, and NLR will be further assessed in forthcoming clinical investigations.
